# Case report: A novel approach of closed-loop brain stimulation combined with robot gait training in post-stroke gait disturbance

**DOI:** 10.3389/fnhum.2023.1082556

**Published:** 2023-01-27

**Authors:** Atsushi Shima, Tomoaki Miyake, Kazuki Tanaka, Akari Ogawa, Erika Omae, Yui Nagamori, Yusuke Miyata, Koji Ohata, Takakuni Maki, Yumie Ono, Tatsuya Mima, Ryosuke Takahashi, Satoko Koganemaru

**Affiliations:** ^1^Department of Regenerative Systems Neuroscience, Human Brain Research Center, Kyoto University Graduate School of Medicine, Kyoto, Japan; ^2^Department of Neurology, Kyoto University Graduate School of Medicine, Kyoto, Japan; ^3^Department of Human Health Sciences, Kyoto University Graduate School of Medicine, Kyoto, Japan; ^4^Department of Neuroscience, Kyoto University Graduate School of Medicine, Kyoto, Japan; ^5^Department of Electronics and Bioinformatics, Meiji University, Kanagawa, Japan; ^6^The Graduate School of Core Ethics and Frontier Sciences, Ritsumeikan University, Kyoto, Japan; ^7^Department of Rehabilitation Medicine, Hokkaido University Hospital, Hokkaido, Japan

**Keywords:** non-invasive brain stimulation, closed-loop stimulation, rehabilitation, gait, robot

## Abstract

Most post-stroke patients have long-lasting gait disturbances that reduce their daily activities. They often show impaired hip and knee joint flexion and ankle dorsiflexion of the lower limbs during the swing phase of gait, which is controlled by the corticospinal tract from the primary motor cortex (M1). Recently, we reported that gait-synchronized closed-loop brain stimulation targeting swing phase-related activity in the affected M1 can improve gait function in post-stroke patients. Subsequently, a gait-training robot (Orthobot^®^) was developed that could assist lower-limb joint movements during the swing phase of gait. Therefore, we investigated whether gait-synchronized closed-loop brain stimulation combined with robot-assisted training targeting the swing phase could enhance the recovery of post-stroke gait disturbance. A 57-year-old female patient with chronic post-stroke hemiparesis underwent closed-loop brain stimulation combined with robot-assisted training for 10 min 2 years after left pons infarction. For closed-loop brain stimulation, we used transcranial oscillatory electrical current stimulation over the lesioned M1 foot area with 1.5 mA of DC offset and 0–3 mA of sine-wave formed currents triggered by the paretic heel contact to set the maximum current just before the swing phase (intervention A; two times repeated, A1 and A2). According to the N-of-1 study design, we also performed sham stimulation (intervention B) and control stimulation not targeting the swing phase (intervention C) combined with robot-assisted training in the order of A1-B-A2-C interventions. As a result, we found larger improvements in gait speed, the Timed Up and Go test result, and muscle strength after the A1 and A2 interventions than after the B and C interventions. After confirming the short-term effects, we performed an additional long-term intervention twice a week for 5 weeks, for a total of 10 sessions. Gait parameters also largely improved after long-term intervention. Gait-synchronized closed-loop brain stimulation combined with robot-assisted training targeting the swing phase of gait may promote the recovery of gait function in post-stroke patients. Further studies with a larger number of patients are necessary.

## 1. Introduction

Gait disturbance in post-stroke patients is a severe impairment that lowers the daily activities of life (Jørgensen et al., 1995; Duncan et al., 2005). It lowers the frequency of mobility, especially for community ambulation, and restricts life-space mobility at 89% compared with healthy individuals. Previous research demonstrated patients and caregivers exhibit increased difficulty in mobility with impaired motivation for ambulation related to the need for assistance (Lord et al., 2004; Tashiro et al., 2019). Moreover, it leads to the difficulty in transferring and going up and down the stairs, and the decreased quality of standing/sitting with increased time to perform (Chou et al., 2003; Lord et al., 2004; de Rooij et al., 2021). Patients often show inadequate flexion of the paretic lower limb during the swing phase with knee hyperextension, called “back-knee” in the initial stance phase due to compensation of hip extensors for the weakness of the quadriceps and anterior tibialis (TA) muscles during the terminal swing and initial stance phase. The knee hyperextension causes high tension on the anterior cruciate ligament and posterior part of the knee in the paretic limb (Kramers et al., 1996; Mulroy et al., 2003; Bleyenheuft et al., 2010; Perry and Burnfield, 2010; Appasamy et al., 2015). During the swing phase, knee flexion and ankle dorsiflexion are reduced with inadequate outputs of the related muscles. Circumduction gait is secondarily produced for maintaining foot clearance in compensation for inadequate flexion of the lower limb (Perry and Burnfield, 2010; Wang et al., 2017). As a probable neuronal mechanism, it is considered that the swing phase-related corticospinal activity is impaired in the contralateral flexor muscles, such as the biceps femoris and TA muscles (Capaday et al., 1999; Yang and Gorassini, 2006; Petersen et al., 2012; Artoni et al., 2017; Kitatani et al., 2020).

Recently, we reported that gait-synchronized closed-loop brain stimulation targeting the swing phase improves post-stroke gait and balance function using transcranial alternating current stimulation (tACS) with a positive DC offset applied over the foot area of the affected primary motor cortex (M1) (Koganemaru et al., 2019; Kitatani et al., 2020). It could specifically improve joint flexion during the swing phase, suggesting that it may have enhanced the swing phase-related M1 activity on the affected side, which was related to the flexor function of the paretic lower limbs during the swing phase. In parallel with our findings, a gait rehabilitation robot (Orthobot^®^) that assists knee joint movements during the swing phase has been developed for post-stroke patients with gait disturbances (Kawasaki et al., 2017). The combination of rehabilitation with brain stimulation has been effective in enhancing specific neuronal networks and functional recovery in neurological patients (Koganemaru et al., 2015) although some reports using a combination of brain stimulation and robotic rehabilitation showed negative results for additional effects (Kumru et al., 2016; Leon et al., 2017). Therefore, we investigated whether closed-loop brain stimulation combined with robot-assisted training could enhance the recovery from post-stroke gait disturbance in a chronic post-stroke hemiparetic patient.

## 2. Case description

### 2.1. Patient characteristics

A 57-year-old woman with post-stroke hemiparesis, dysarthria, and difficulty walking was referred to a university hospital. At 55 years of age, she was diagnosed with cerebral infarction in the left pons, left cerebral peduncle, and both cerebellums, corresponding to the territory of the anterior inferior, posterior inferior, and superior cerebellar arteries (Table 1). Her comorbidities were diabetes mellitus without diabetic neuropathy or hypothyroidism after thyroid goiter resection.

**Table 1:** Clinical findings of the case.

**Months**	**Clinical findings**
0	Admission to the hospital for acute onset of right vertigo and difficulty in hearing followed by dysarthria and right hemiparesis. •Magnetic resonance imaging revealed acute cerebral infarction of left pons, left cerebellar, peduncle, bilateral cerebellum.•Suspected as artery to artery embolism from the stenosis of posterior circulations.
1.5	Transferred to the rehabilitation hospital.
7.5	Discharged from the hospital. •Right hemiparesis and difficulty in walking, dysarthria remained.
23	Participated in the study.

### 2.2. Therapeutic intervention

We conducted three types of interventions according to an N-of-1 study design: (1) tACS with a positive DC offset to the lesioned M1 foot area targeting the swing phase (intervention A), (2) sham stimulation (intervention B), and (3) tACS with the same parameters but not targeting the swing phase (intervention C). All stimulations were combined with a 10-min robot-assisted training using Orthobot^®^ (Integra Inc.), which assists knee joint movements during the swing phase by sensing the posture angle of the femur of the supported lower limb (Kawasaki et al., 2017). KineAssist with truncal belts (Woodway USA, Inc.) was used to prevent falls.

Before the intervention, the gait cycle was measured using a 30-second treadmill gait at a self-paced speed. For tACS, an anodal electrode was placed on the left M1 foot area (3 × 3 cm), and the location of the stimulation was determined using the hotspot of the affected TA muscles, which produced the largest motor evoked potentials of the TA muscles by transcranial magnetic stimulation. A cathodal electrode (7 × 5 cm) was placed on the contralateral shoulder. The electrical current of tACS was a sinusoidal wave of 3-mA peak-to-peak amplitude (~0–3 mA) with 1.5 mA of the positive DC offset computed with an external computer, and it was applied to the DC stimulator (NeuroConn DC, GmbH) according to a previous report (Koganemaru et al., 2019).

In intervention A (tACS targeting the swing phase), the current cycle started from 0 mA at the time of floor contact of the paretic right heel detected by the pressure sensor pasted on the heel to make the peak of the current intensity match just before the initiation of the swing phase of the right paretic lower limb. In intervention B (sham stimulation), 10 cycles of electrical currents were produced with the same electrode montages. In intervention C, the cycles of the current started with a delay of 0.5 gait cycle at the time of floor contact of the right heel, and the peak of the current intensity was around the initiation of the stance phase, not targeting the swing phase. The order of the interventions was A (A1), B, A (A2), and C. Each short-term intervention was performed separately, and the interval between the sessions was over 1 week.

In addition, we performed a long-term intervention of repeated interventions A twice a week for 5 weeks for a total of 10 times. In the long-term intervention, we set the speed of the treadmill and the frequency of tACS using a 30-second gait before every session. We checked the impedance of the electrodes before stimulation as below 5 kohm, and asked the participants whether she has phosphene and cutaneous sensations for each session.

### 2.3. Clinical measurements

#### 2.3.1. Short-term evaluations

Clinical evaluations were performed before and immediately after the short-term interventions (interventions A, B, and C) on the same day. We measured the velocity and number of steps in the 10-m walk test at a comfortable speed (10 m CS gait) and at a maximal speed (10 m MS gait) and conducted the Timed Up and Go test (TUG). A walker was used in the 10-m walk and TUG test.

#### 2.3.2. Long-term follow-ups

Clinical evaluations were performed before, immediately after, and 2 weeks after the long-term interventions. We measured the velocity and number of 10 m CS and MS gaits and determined results of the TUG tests using a caster walker, modified Ashworth Scale [MAS (Bohannon and Smith, 1987)] in the paretic hamstring and triceps surae, Fugl-Meyer Assessment of Lower Extremity (FMA-LE, Fugl-Meyer, 1980), and Mini-Balance Evaluation Systems Test (mini-BESTest, Horak et al., 2009).

### 2.4. Analysis of gait kinematics of the lower limbs

To differentiate the effects of the combined brain stimulation on gait kinematics, we measured the joint angles of the lower limbs in the 5.5-m walk on level ground using the three-dimensional (3D)-gait analysis system (Noraxon *MyoMOTION*^TM^, myoMuscle Master Edition System) before and after each short-term intervention (interventions A1, B, A2, and C). Motion capture sensors were placed on both femurs, tibias, and dorsum of the feet at a sampling frequency of 100 Hz, and 3D-gait analysis was performed.

We assessed limb segment angle covariation, which is considered to indicate neuronal control of the coordination of limb segments during human gait (Borghese, Bianchi, and Lacquaniti 1996; Ivanenko et al., 2007). Owing to the particular pattern of time-dependent activation of muscle synergies, kinematic synergies are produced by the coordinated rotations of the ankle, knee, and hip joints during human gait (Ivanenko et al., 2007). The temporal changes in the elevation angles of the lower limb segments are tightly coupled, and regular trajectory loops are constrained close to a plane when the elevation angles are plotted against one another (Ivanenko et al., 2007). Based on previous studies, we calculated the ensemble averages of the femur (greater trochanter-knee), tibia (knee-ankle), and foot elevation angles (ankle-third metatarsus) of both legs, evaluated the corresponding trajectories in the segment angle space onto the covariation plane (CVP), and performed principal component analysis (Borghese et al., 1996; Ivanenko et al., 2007; Degelean et al., 2012). In normal human gait, the first (PC1) and second (PC2) components are supposed to lie on a plane of angular covariation (intersegmental CVP) and form a 2-dimensional time-dependent path as the gait loop with an ellipse-like shape. The sum of the variance of PC1 and PC2 has been reported to explain >99% of the total variance, indicating the planarity of intersegmental coordination during gait (Ivanenko et al., 2007). Then, we assessed the sum of the variance of the PC1 and PC2 as a planarity index to reflex the intersegmental coordination in each side of the lower limbs (planarity), and the differences of areas of the gait loop (GLA) between the paretic and non-paretic lower limbs [= 2 × (GLA _*paretic*_ – GLA _*non*−*paretic*_)/(GLA _*paretic*_ + GLA _*non*−*paretic*_)] as an asymmetry index of the lower limb segmental coordination during gait (GLA asymmetry) before and after each short-term intervention.

### 2.5. Outcomes

No adverse and unanticipated events were developed during the interventions. The patient did not feel any sensation such as phosphenes and cutaneous irritation.

#### 2.5.1. Short-term interventions

**Figure 1:**
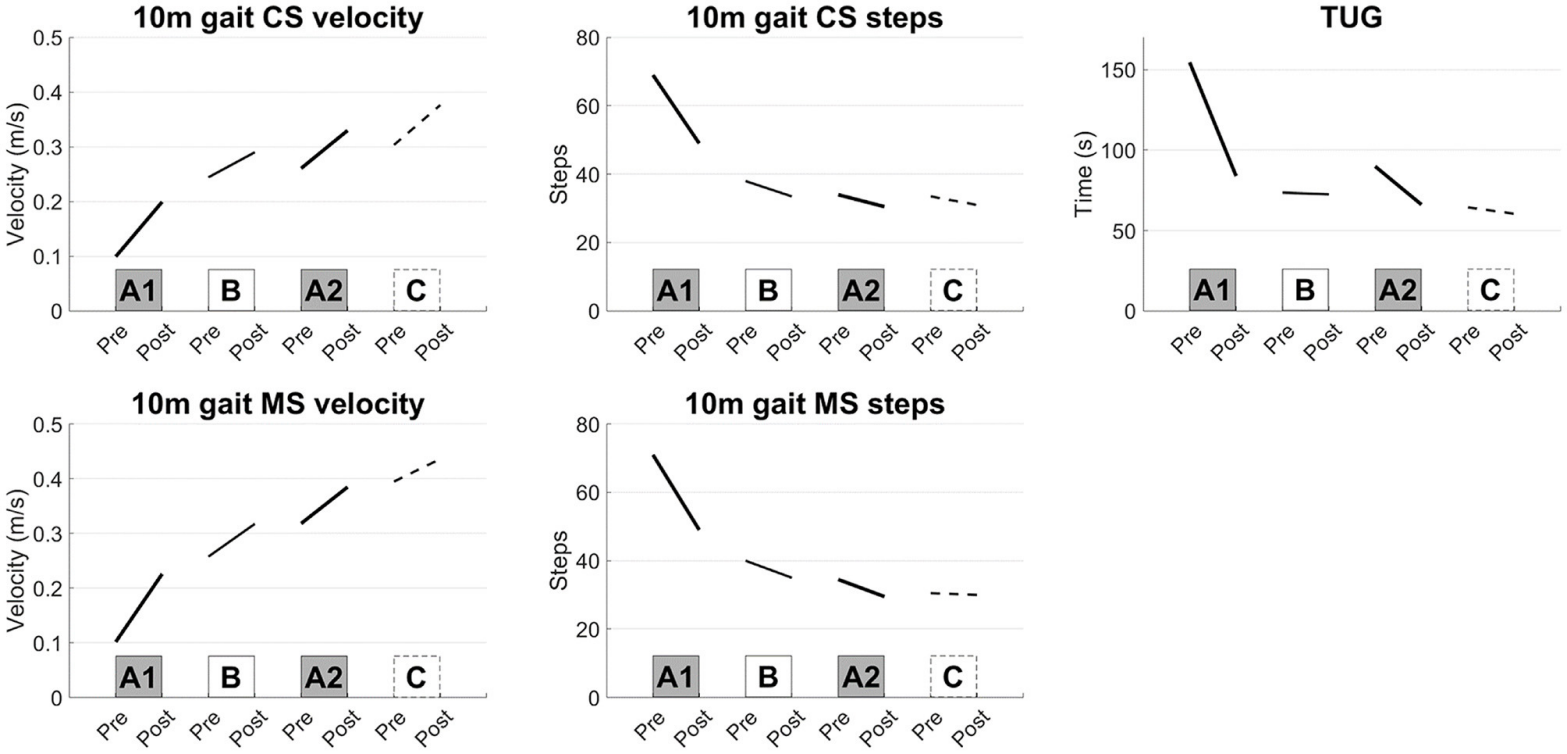
Results of the short-term evaluations. Synchronized stimulation improved the gait speed measured by the 10-m gait test, TUG test, and muscle strength of the lower limbs (interventions A1 and A2), whereas sham or asynchronized stimulation did not improve the gait speed (interventions B and C). 10 m gait CS: 10-m walk test at a comfortable speed, 10 m gait MS: 10-m walk test at a maximal speed, TUG: Timed Up and Go test.

**Figure 2:**
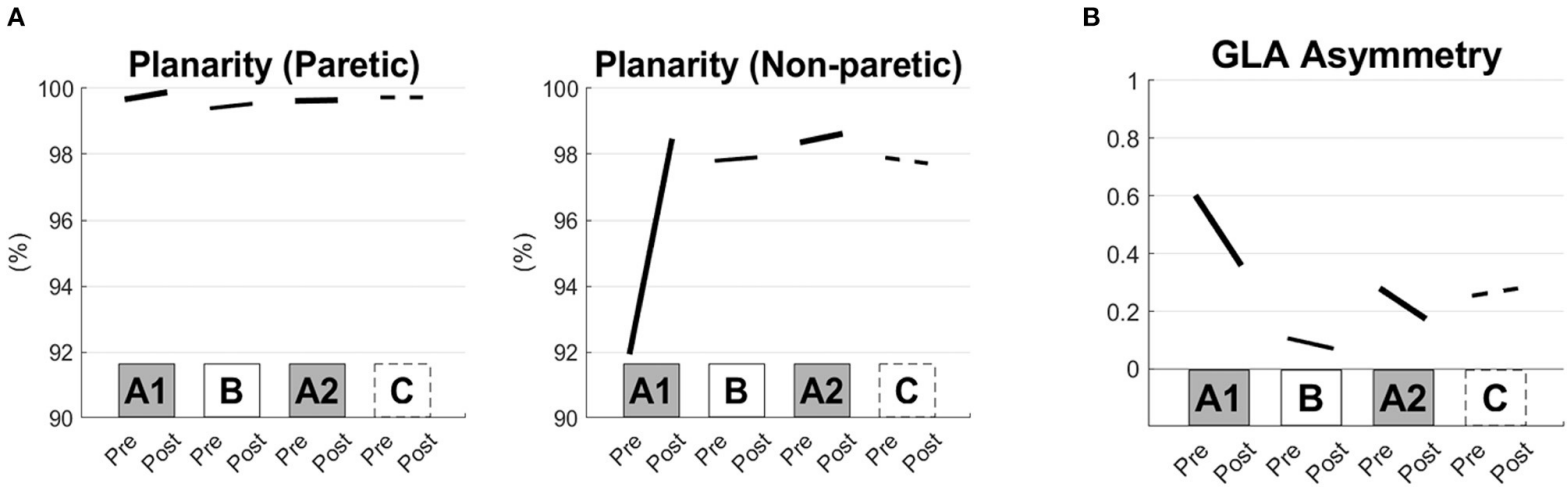
Kinematic gait parameters before and after the short-term interventions. Planarity of the paretic and non-paretic sides of the lower limbs **(A)** and areas of the gait loop (GLA) asymmetry **(B)** are shown before and after each of the interventions (the pre- and post-conditions of the A1, B, A2, and C interventions).

The frequency of the tACS was set at 0.57 ± 0.28 Hz (mean ± S.D.). Large improvements were induced in the 10 m CS gait velocity, step length, result of TUG and muscle strength of the lower limbs after interventions A1 and A2 compared with those after interventions B and C (Figure 1). The kinematic parameters showed the largest improvements in planarity on the non-paretic side after the A1 and A2 interventions (Figures 2A, B).

#### 2.5.2. Long-term follow-ups

**Figure 3:**
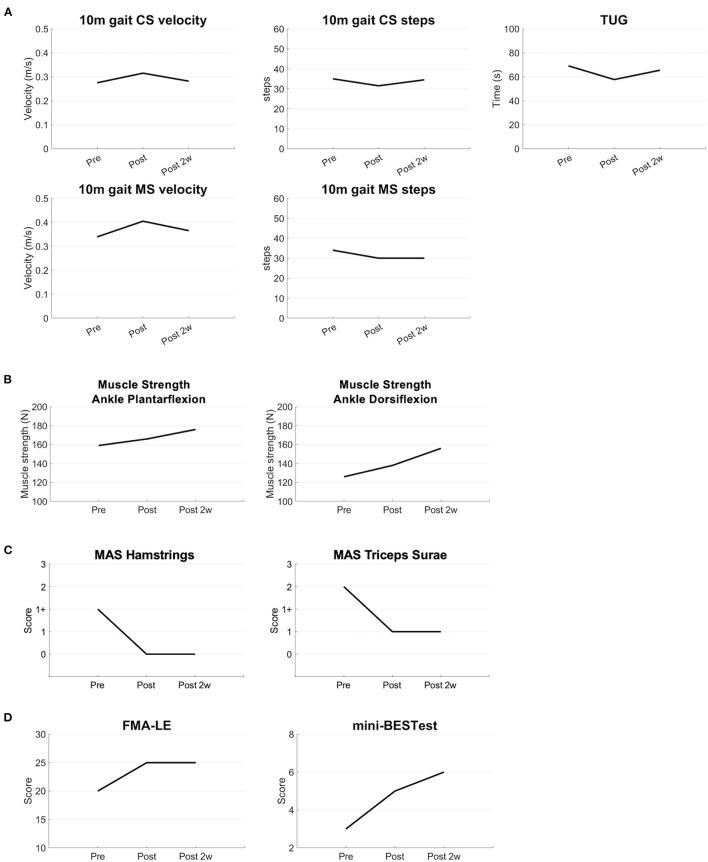
Results of the long-term evaluations. The clinical parameters are shown before, immediately after, and 2 weeks after the intervention **(A)** The results of the 10 m CS and MS gait tests show an increased velocity, decrease of the number of steps, and decrease of the time of the TUG test **(B)** Muscle strength of the ankle joint is increased after the intervention **(C)** MAS scores are lower after the intervention **(D)** FMA and mini-BESTest scores are improved after the intervention. CS, comfortable speed; MS, maximal speed; TUG, Timed Up and Go; MAS, modified Ashworth scale; FMA-LE, Fugl-Meyer Assessment Lower Extremity; mini-BESTest, mini-Balance Evaluation Systems Test.

The additional long-term follow-ups of the synchronized brain stimulation with robot-assisted training increased gait velocity, decreased the number of steps in the 10-m gait test at the comfortable speed and maximal speed, and shortened the time measured by the TUG test, suggesting that gait and balance function were improved (Figure 3A). Muscle strength also increased in plantar flexion and dorsiflexion of the affected ankle (Figure 3B). The spasticity measured by the MAS was decreased in the hamstring and triceps surae of the affected side (Figure 3C). The FMA and mini-BESTest scores increased, suggesting improvements in general motor and balance functions (Figure 3D). The improvements persisted for at least 2 weeks after the intervention.

## 3. Discussion

The present patient with post-stroke gait disturbance showed functional improvements in gait after closed-loop brain stimulation targeting the swing phase combined with the gait robot to assist limb movements during the swing phase. She showed improvements in gait speed and step length measured by the 10-m gait and TUG tests after synchronized brain stimulation combined with robot-assisted training, but not after sham and asynchronized brain stimulation.

Robot-assisted rehabilitation is an overwhelmingly growing field with technical improvements. Robot-assisted rehabilitation approaches vary in terms of physical implementation, interaction, and targeting of sensorimotor pathways (Hobbs and Artemiadis, 2020). This seems to have a positive effect on walking independence. However, the effect of robot-assisted training on detailed gait parameters, such as velocity, has not yet been elucidated (Mehrholz et al., 2013).

Combined approaches for chronic post-stroke patients with robot assistance for gait ability and non-invasive brain stimulation have been reported to possibly have a positive effect on gait function (Bressi et al., 2022). However, the results have been inconclusive. A recent study on robot gait training combined with anodal tDCS over the vertex and the reference electrode on the supra-orbital region showed an improvement in the distance measured by the 6-min walking test at 4 weeks after the treatment (Seo et al., 2017) and in the functional ambulation category in a small number of chronic post-stroke patients (Danzl et al., 2013). In contrast, another report showed no significant effect of anodal tDCS over the motor area controlling the leg with the reference electrode on the contralateral supra-orbital region combined with robot training compared with robot training alone (Geroin et al., 2011). The inconclusive results suggest that a more specific protocol for brain stimulation parameters and robotic treatment may be appropriate for post-stroke gait disturbance.

To target the specific gait impairment of chronic post-stroke patients, we focused on the swing phase showing impaired hip and knee joint flexion and ankle dorsiflexion, leading to a lower foot clearance with a decrease in gait speed (Chen et al., 2005). Among the various types of robots, a recently developed robot-assisted training using a device for controlling knee flexion and extension during the swing phase is effective in improving the gait functions of post-stroke patients immediately after a single session of robot-assisted training (Kawasaki et al., 2017). tACS over the M1 foot area entrained the gait cycle to enhance swing phase-related M1 activation in healthy individuals (Koganemaru et al., 2018; Kitatani et al., 2020), and closed-loop gait synchronized tACS targeting the swing phase of the paretic leg induced synaptic plasticity in swing phase-related M1 activities and improved gait and balance performance in chronic post-stroke patients (Koganemaru et al., 2019). A combination of both approaches was effective in the present case. The synchronized brain stimulation with robot-assisted training targeting the swing phase could improve the gait speed measured by the 10-m gait test, TUG test, and muscle strength of the lower limbs, compared with the robot-assisted training alone and stimulation without targeting the swing phase with robot-assisted training.

The present case demonstrated the improvements of the TUG test. The TUG test can assess the balance function as well as gait speed (Shimada et al., 2010; Lopes et al., 2015). Previous research suggested that the balance function was correlated with the swing-phase parameters leading to the larger step width (Lee et al., 2021). Therefore, the improvements of the temporal control during the swing phase may have also improved the balance function and the TUG test.

Kinematic alteration of the gait pattern was induced in both paretic and intact lower limbs, especially after the first intervention of the combined synchronized brain stimulation with robot-assisted training targeting the swing phase. Increased planarity means that the deviant kinematic gait pattern returned to normal, especially in the intact lower limb. In addition, the asymmetry of the kinematic patterns of both legs was reduced, leading to recovery of gait speed (Ivanenko et al., 2007).

In human bipedal gait, phasic corticospinal inputs have been identified dominantly during the swing phase according to the gait cycle and the activity of M1 area showed the gait-cycle dependent dynamics (Capaday et al., 1999; Yang and Gorassini, 2006; Petersen et al., 2012; Artoni et al., 2017). In the previous studies, tACS with the frequency matched with the gait cycle showed the enhancement of gait-dependent phasic activity of the lesioned M1 in post-stroke patients (Koganemaru et al., 2019; Kitatani et al., 2020). Therefore, we have stimulated the lesioned M1 with the frequency matched with the gait cycle of the paretic lower limb in this case and found its effectiveness. We used the different intensity of tACS and reference electrode position from those of the previous study (Koganemaru et al., 2019) because the present parameters showed less variability and tACS-associated perception such as dizziness in the preliminary assessments of healthy subjects (unpublished data). Meanwhile, gait function is related to activities of the other cortical areas such as supplemental motor and premotor areas than the M1 (Fukuyama et al., 1997; Iseki et al., 2010; Takakusaki, 2017). Further comparative study would be necessary by tACS with other frequencies and different cortical areas.

In the present case, the participant used the walker in the assessment of gait parameters. Walkers are reported to increase gait speed (Riley et al., 2007; Cetin et al., 2010). Therefore, it would possibly affect the patient's gait speed although we could not assess it because she could not walk without a walker. Further assessment would be necessary to clarify the influence by comparing with gait without walking aids. We performed the intervention using the self-paced treadmill. In previous reports, there was no significant difference of gait cycle speed variability and symmetry between the self-paced treadmill and overground gait (Holmes et al., 2021; Van et al., 2022), although the fixed-speed treadmill had an influence on the preferred gait speed and kinematic angles (Riley et al., 2007; Cetin et al., 2010; Malatesta et al., 2017). It is considered that the self-paced treadmill may have had little influence on the temporal control of gait parameters during the intervention compared with over ground gait in this case. However, further investigation would be necessary to validate the influence of the treadmill during the intervention.

The present case of chronic phase after stroke revealed the possibility of restoring gait function after stroke with a novel combination of closed-loop synchronized brain stimulation and robot-assisted training. The results from the additional long-term follow-ups showed the maintained gait and balance functions measured by the mini-BESTest, FMA, and TUG test for at least 2 weeks after the interventions. The clinical phase after stroke affects the outcome of the disabilities (Li et al., 2018; Ballester et al., 2019). In the acute or subacute phases, the remodeling process occurs *via* neurogenesis and gliogenesis, leading to more favorable outcomes through rehabilitative training (Li et al., 2018; Ballester et al., 2019; Cirillo et al., 2020). Therefore, the present approach may bring more improvements in the acute or subacute phase. Meanwhile, the present case demonstrated that the current approach enabled functional restoration even in the chronic phase. Longitudinal studies are warranted for more cases in the different post-stroke phases to elucidate an appropriate clinical phase for the current approach.

## Author's note

The patient stated that she hoped that the present findings will contribute to new approaches to improve disabilities caused by stroke.

## Data availability statement

The raw data supporting the conclusions of this article will be made available by the authors, without undue reservation.

## Ethics statement

The studies involving human participants were reviewed and approved by the Hokkaido University Certified Review Board. The patient provided her written informed consent to participate in this study. Written informed consent was obtained from the patient for the publication of any potentially identifiable images or data included in this article.

## Author contributions

AS, ToM, and SK designed the study and collected and interpreted the data. AS and SK contributed to the initial draft of the manuscript. AS, ToM, AO, YN, YM, EO, TaM, KO, and YO contributed to data collection and analysis. RT and TatM reviewed the manuscript critically. All authors approved the final version of the manuscript.
